# T Cells With Activated *STAT4* Drive the High-Risk Rejection State to Renal Allograft Failure After Kidney Transplantation

**DOI:** 10.3389/fimmu.2022.895762

**Published:** 2022-07-01

**Authors:** Yihan Chen, Bao Zhang, Tianliang Liu, Xiaoping Chen, Yaning Wang, Hongbo Zhang

**Affiliations:** ^1^ Key Laboratory for Stem Cells and Tissue Engineering, Ministry of Education, Sun Yat-sen University, Guangzhou, China; ^2^ Advanced Medical Technology Center, The First Affiliated Hospital, Zhongshan School of Medicine, Sun Yat-sen University, Guangzhou, China; ^3^ The Department of Histology and Embryology, Zhongshan School of Medicine, Sun Yat-sen University, Guangzhou, China

**Keywords:** kidney transplantation rejection, transcriptome-based re-classification, high-risk rejection, T cells, *PTPN6-STAT4* signaling

## Abstract

In kidney transplantation, deteriorated progression of rejection is considered to be a leading course of postoperative mortality. However, the conventional histologic diagnosis is limited in reading the rejection status at the molecular level, thereby triggering mismatched pathogenesis with clinical phenotypes. Here, by applying uniform manifold approximation and projection and Leiden algorithms to 2,611 publicly available microarray datasets of renal transplantation, we uncovered six rejection states with corresponding signature genes and revealed a high-risk (HR) state that was essential in promoting allograft loss. By identifying cell populations from single-cell RNA sequencing data that were associated with the six rejection states, we identified a T-cell population to be the pathogenesis-triggering cells associated with the HR rejection state. Additionally, by constructing gene regulatory networks, we identified that activated *STAT4*, as a core transcription factor that was regulated by *PTPN6* in T cells, was closely linked to poor allograft function and prognosis. Taken together, our study provides a novel strategy to help with the precise diagnosis of kidney allograft rejection progression, which is powerful in investigating the underlying molecular pathogenesis, and therefore, for further clinical intervention.

## Introduction

Kidney transplantation is the gold standard treatment for most patients with end-stage kidney disease (ESKD), whereas transplantation rejection leads to allograft loss (1). Despite the widely accepted histology-dependent criteria Banff for the diagnosis of rejection progression, it is still limited to precisely distinguish different graft rejection statuses due to the inherent requirement for subjective assessment (2). Moreover, histology-basis assessment makes it impossible to identify pathogenesis at the molecular level, especially on the immune aspect, which triggers an increase in inflammatory burden to allograft function (3–7). These above limitations altogether leave an urgent concern to obtain appropriate strategies for a better diagnosis and for molecular pathogenesis investigation.

Bulk transcriptomic datasets from renal transplantation biopsies have been applied to reveal rejection states in a more accurate way. Based on mechanical learning algorisms (2) and signature gene sets regarding rejection status (8–10), these studies have established prognostic models to refine the traditional clinical classifications. However, it is still hard to meet the needs of repetitive and systematic classification on different rejection states, and it is difficult to further understand the underlying molecular mechanisms driving transplant rejection.

The recently developed single-cell RNA sequencing (scRNA-seq) allows the measurement of transcriptomes from individual cells, which provide new insights into complex biological systems and enable the identification of rare cell types, new cell states, as well as intercellular communication networks that may be masked by traditional bulk transcriptional profiling (11). It also offers an unprecedented opportunity to define cell types and states comprehensively with molecular precision (12). Therefore, combination data taken from the advancement of both scRNA-seq and bulk transcriptome strategies would be helpful to discover special cell types and heterogeneous gene signatures of rejection subpopulations in response to different stages of rejection.

In this study, we establish a new pathogenic classification of renal allograft rejection status based on Uniform Manifold Approximation and Projection (UMAP) and Leiden algorithms at single-cell resolution. Using this strategy, we identified a high-risk (HR) rejection status prone to allograft loss, which was mediated by accumulated T-cell immune responses. Through constructing gene regulatory networks, we further uncovered a *PTPN6*-involved and *STAT4*-dominated mechanism, which provides new insights for clinical interventions for renal allograft failure.

## Materials and Methods

### Data Collection

A total of 2,611 human microarray datasets from tissue biopsies of kidney allografts were obtained from the Gene Expression Omnibus (GEO) database (**Supplementary Table 1**). According to the Banff standard, samples diagnosed as “non-rejection” are classified as “stable state (STA)”, and samples diagnosed as “antibody-mediated rejection (ABMR)” and “T-cell mediated rejection (TCMR)” are classified as “Mixed state (Mix)”. All other data diagnosed as “borderline” are excluded. The data used are all samples from patients diagnosed as ABMR, TCMR, acute rejection (AR), stable state (STA), chronic rejection (CR), and mixed state (Mix) (2, 3, 8, 13–21). The single-cell datasets of samples were downloaded from GEO (12, 22) (GSE145927 and GSE109564), which were collected from kidney biopsies with a diagnosis of acute ABMR and acute Mix.

### Microarray Data Preprocessing

Microarray datasets were re-annotated to unify the gene names corresponding to each probe. We aligned all probe sequences from 2,611 microarray datasets to the FASTA file of hg38 using bowtie2 (23) and annotated them using bedtools (24). The samples from each series record of GEO were standardized using limma (25). Two expression matrices were prepared. The first one was acquired by directly merging the matrices from each GSE based on the intersection of genes without removing the batch effect and normalization. The second matrix was progressed with Combat to remove batch effects. Batch effect was removed according to the GPL number and the company category. All the combined data of the second matrix were normalized by the logarithm of 2.

### Single-Cell Transcriptomic Sequencing Data Preprocessing

The raw gene expression matrices of scRNA-seq datasets from all renal samples were merged and converted to an Anndata object using the Python package Scanpy (version 1.4.4) (26). Cells that expressed less than 500 genes and genes detected in less than 3 cells were filtered out. Potential doublet cells were then detected and filtered by applying the Python package scrublet (version 0.2) (27) for each sample. Next, doublet-dominated sub-clusters were checked to ensure a low doublet rate in all populations using the previously described method (28). The gene expression levels were normalized by the total UMI count per cell (1e4) with data being log-transformed. The interferences arising from cell cycling genes were eliminated by using the regress_out function of the Scanpy package. Then, highly variable genes (HVGs) in gene expression matrices were identified for further analysis using the highly_variable_genes function of the Scanpy package. Finally, the batch effect was eliminated using the Python package bbknn (version 1.2.0) (29). The dimensionality of HVGs was primarily reduced by principal component analysis (PCA). The first 40 principal components were further summarized by UMAP for dimensionality reduction using the default setting of the UMAP function of the Scanpy package. Cells were clustered with the Leiden algorithm using the leiden function of the Scanpy package. Cell-specific gene markers across all cell types were identified with the get_DEG_single function of Python package PLOGS (https://github.com/ZhangHongbo-Lab/PLOGS) that we developed, with parameter ratio ≥ 0.5 and *q*-value ≤ 1e-30.

### Reclassification Based on Dimensionality Reduction and Clustering Algorithm

To reclassify the samples of human microarray datasets, we used Scanpy (version 1.6.0) to read the first expression value data and the log1p function of Scanpy to perform logarithmic calculations. Then, HVGs were calculated based on each batch and screened with parameter batches ≥ 1. The second expression value data was screened using the HVG, which was the third expression matrix. The dimensionality of HVGs was primarily reduced by PCA. The first 40 principal components were further summarized by UMAP (30) dimensionality reduction using the default setting of the umap function of the Scanpy package and clustered with the Leiden algorithm using the leiden function of the Scanpy package. In order to make the data classification results clearer and more credible, the batch effect was eliminated again using the Python package bbknn (version 1.2.0). The cluster-specific gene markers were identified with the get_DEG_single function of the Python package PLOGS with parameter ratio ≥ 0.5 and *q*-value ≤ 1e-30. The reclassified clusters were annotated by differential expression gene groups of six rejection states.

### Identification of Rejection State Associated Cells

Scissor (31) was performed to identify the cell subpopulations most highly associated with the states of reclassified clusters in bulk RNA-seq data. All of the states of reclassified clusters were merged, and each cell corresponded to the rejection state with the largest positive correlation coefficient.

### Prediction of the Proportion of Cell Types and Capture of Important Cell Types

The expression matrices of the marker genes of each cell type were regarded as the cell characteristics, and CIBERSORT (32) was performed to analyze the cell ratio of the six states using the 2,611 microarray datasets. The R package pheatmap was used to visualize the results, and the cell type was set to scale for horizontal comparison. Then, we output the numerical matrix after the scale, calculated the power function matrix corresponding to the numerical matrix with 2 as the base, and rounded it, selecting the cell type with the value greater than 1 as the potentially important cell type. This value was used as the proportion of the cell type in the corresponding rejection state. The score_genes function of the Scanpy package was used to show the degree of gene expression in different states.

### Co-Expression Network Construction

Co-expression network analysis was performed using weighted correlation network analysis (WGCNA, R package) (33). All genes were selected as input matrix. The co-expression network was constructed by the automatic construction function with the parameter power 10. Co-expression network was visualized by Cytoscape (34). IRegulon (35) was used to predict the transcription factors.

### Upstream Network Analysis in T Cells

NicheNet (36) was used to predict upstream regulatory networks that drive *STAT4*. All expressed genes in T cells were used as the background of genes. Genes were considered as expressed when they have nonzero values in at least 10% of the cells in a cell type. Here, all ligands were adopted to determine signaling paths between ligands and *STAT4*.

### Establishment and Assessment of Predictive Models

Putative STAT4-regulated genes excluding those with insignificant correlations were applied to least absolute shrinkage and selection operator (LASSO) regression to identify critical prognostic genes and construct a diagnostic model with low variance and strong universality. The dataset GSE21374 was divided into training and validation sets by 6:4 randomization without replacement. The package glmnet was used in R version 4.1.0.

## Results

### Unsupervised Clustering Reveals a High-Risk Status Prone to Renal Allograft Failure

In the current clinical diagnosis of kidney transplant prognosis, allograft rejection is usually classified into six progression statuses based on divergent histology manifestations: stable (STA), antibody-mediated (ABMR), acute (AR), chronic (CR), T-cell mediated (TCMR), and mixed TCMR with ABMR (Mix) rejection. To evaluate the correlation between state from current clinical diagnosis and transcriptome and understand the cellular and molecular mechanisms that lead to kidney transplant rejection, we first applied the current clinical criteria and analyzed microarray data of 2,611 kidney biopsies (2, 3, 8, 13–21) from patients receiving renal transplantation (**Supplementary Table 1**). Through PCA, we found that allograft samples with different clinical diagnoses were mixed and randomly distributed in two separate groups (**Supplementary Figure 1A**), suggesting the discrepancy between traditional clinical diagnosis and transcriptomic heterogeneity of disease status. At the gene expression level, different clinical diagnoses were also difficult to be recognized by differentially expressed genes (DEGs) as compared between each rejection state and the stable condition (**Supplementary Figures 1B, C**). Similar results were also observed in other previous transcriptomic analyses (2, 17, 37). These results indicated that the traditional clinical classifications might not accurately distinguish patients with different rejection states at the molecular level.

**Figure 1:**
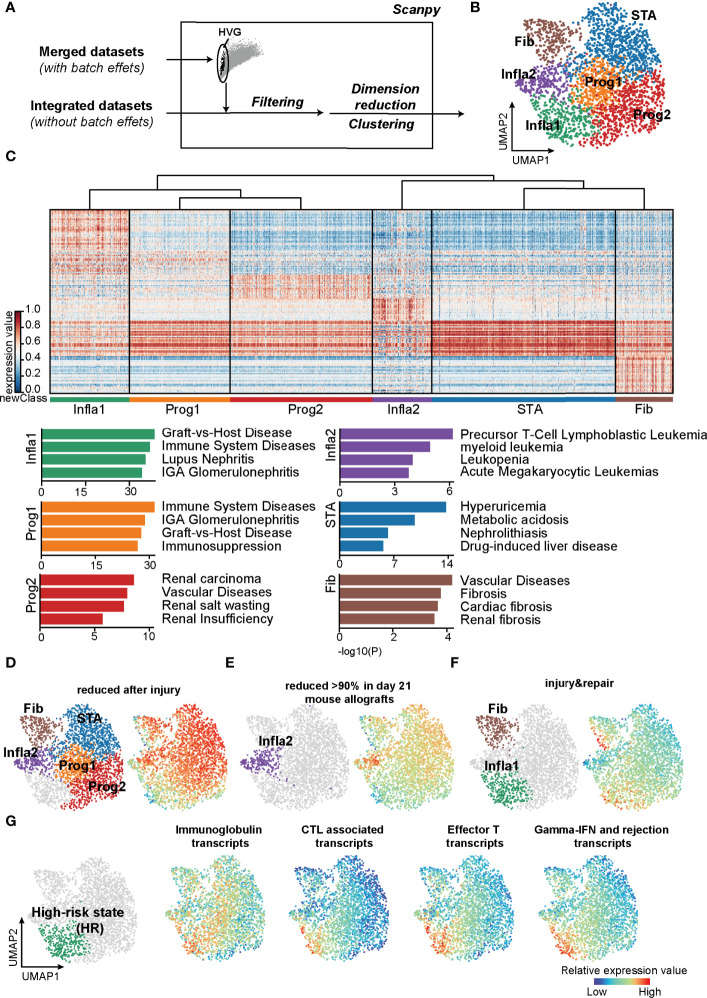
Classifications based on the unsupervised algorithms revealed a high-risk rejection state prone to allograft lost. **(A)** The flowchart of reclassification towards renal transplantation rejection. HVG, highly variable genes. **(B)** UMAP plot showing annotations of the 6 newly defined rejection states. Infla1, inflammatory state 1; Prog1, progressive state 1; Prog2, progressive state2; Infla2, inflammatory state 2; STA, stable state; Fib, fibrosis state. **(C)** Heatmap showing signature genes of each rejection state (left panel) and the enriched disease terms for the corresponding state (right panel). **(D–F)** Transcripts from different rejection phenotypes from mouse allograft datasets were enriched in the corresponding rejection states. **(G)** Enrichment of transcripts presenting high risk of graft failure in Infla1. CTL, cytotoxic T lymphocytes.

To precisely refine the rejection status at the molecular level, we constructed a classification pipeline based on UMAP and Leiden (**Figure 1A**). Unsupervised clustering of 2,611 samples from kidney transplantation yielded 6 main rejection states (**Figure 1B**). As expected, each status was distinguished by its signature gene sets and characterized by specific Gene Ontology (GO) functions (**Figure 1C**; **Supplementary Table 2**). Samples in the stable state (STA) showed significant enrichment in renal homeostasis and detoxification function (**Supplementary Figure 2**) representing a relevant homeostatic condition after receiving transplantation. In addition, samples in fibrosis state (Fib) indicated allografts suffering from fibrotic diseases. The population of inflammatory state 2 (Infla2) seemed to represent a status suffering from dysfunctional leukocytes. With enrichment of tube morphogenesis, renal insufficiency, and humoral immune function, both the progressive state 1 (Prog1) and 2 (Prog2) were characterized to be the activated progression stages of rejection.

Interestingly, the population of inflammatory state 1 (Infla1) was not only correlated with graft-versus-host disease, but also predominantly enriched in immune activation responses with more than 70% of samples in Infla1 showing apparent rejection phenotypes, representing the severest rejection status (**Figure 1C**; **Supplementary Figures 2**, **3**). To further verify its rejection-triggering feature, we mapped these new classifications and phenotypes on human and mouse renal allografts (37) (https://www.ualberta.ca/medicine/institutes-centres-groups/atagc/research/gene-lists, **Supplementary Table 3**). Transcript sets that were reduced after injury or rejection (termed “reduced after injury” and “reduced >90% in day21 mouse allografts”) were enriched in all rejection states that specifically excluded Infla1, suggesting that these states, to some extent, still maintained stability (**Figures 1D, E**). However, the injury- and repair-induced transcripts were highly enriched in Infla1 and, to a lesser extent, Fib, indicating that a part of allograft samples in Fib and Infla1 were damaged (**Figure 1F**).

Previous studies showed that immunoglobins, effective CD8^+^ T cells, and cytotoxic molecules such as GZMB and IFN-γ increase the risk of graft failure (38–42). To identify the group with a high risk of graft failure, we selected transcripts that not only were used in clinical diagnosis but also represented a high risk of allograft loss, and calculated the overall expression scores (**Supplementary Table 3**). Interestingly, all four HR transcript sets showed the highest expression scores in Infla1, further indicating that Infla1 was the HR status prone to allograft loss (**Figure 1G**). Considering all of the above lines of evidence, we identified Infla1 as the HR state.

### T Cells Are Recruited in Triggering HR Rejection

**Figure 2:**
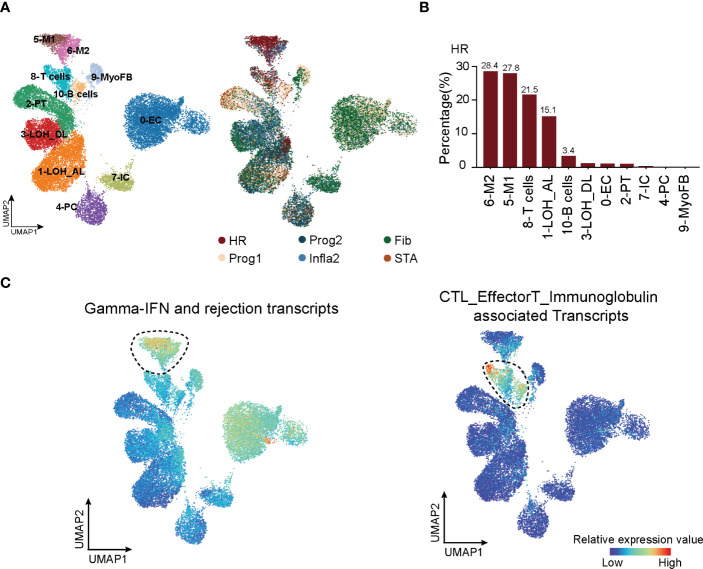
T cells are recruited in triggering HR rejection. **(A)** UMAP plot showing all cell clusters and their annotations in the atlas (left panel) and the corresponding rejection state (right panel). LOH_AL, loop of Henle, ascending limb; LOH_DL, loop of Henle, distal limb; Endo, endothelial cell; PT, proximal tubule; PC, principal cell; MyoFB, myofibroblast; IC, intercalated cell. **(B)** The proportion of various clusters of cells in each rejection state. **(C)** Enrichment of transcripts presenting high risk of graft failure in immune cell types. HR, high-risk state; Prog1, progressive state 1; Prog2, progressive state2; Infla2, inflammatory state 2; STA, stable state; Fib, fibrosis state.

To uncover key cell types with a significant impact on HR state, we first collected scRNA-seq datasets (12, 22) to analyze all cell types present in kidney rejection samples. Unsupervised clustering of the scRNA-seq data from 3 patients identified 11 main cell types defined by signature genes (**Figure 2A**, left panel; **Supplementary Figures 4A, B**; **Supplementary Table 4**). Using the Scissor (31) toolkit, we assigned all cells with each of the rejection states (**Supplementary Figure 4A**) and further applied 23,082 positively relevant cells to better illustrate the relationship between specific cell types and rejection states (**Figure 2A**, right panel). Notably, we identified type I (M1) and type II (M2) macrophages and T cells were strikingly aggregated in HR. Previous knowledge recognized that macrophages and T lymphocytes were the dominant cell types infiltrating acutely rejecting grafts (43). T lymphocytes are central in promoting transplantation rejection and organ damage through allorecognition of foreign antigens and effector responses (44) (**Figure 2B**). We further performed CIBERSORT (32) to predict relative ratios of each cell type in rejection states and also revealed the immune-related cell types including macrophages, T cells, and B cells highly aggregated in HR (**Supplementary Figure 4C**). These results revealed the cell-type characteristics of each rejection state, which were strikingly beneficial for clinical diagnoses. Interestingly, the enrichment of T cells in HR had more significant differences, showing that changes in the amount of T cells were much stronger than other immune cell types in transcriptional datasets (**Supplementary Figure 4C**). Indeed, in further distinguishing cell types highly related to HR by applying HR transcript sets to scRNA-seq, we observed that immune cells including macrophages, T cells, and B cells were enriched in the HR state, while T cells appeared to be specifically involved, indicating that T cells were more recruited in driving HR rejection progression (**Figure 2C**).

### *STAT4* Is Essential in Mediating T-Cell Immune Responses in HR Rejection

**Figure 3:**
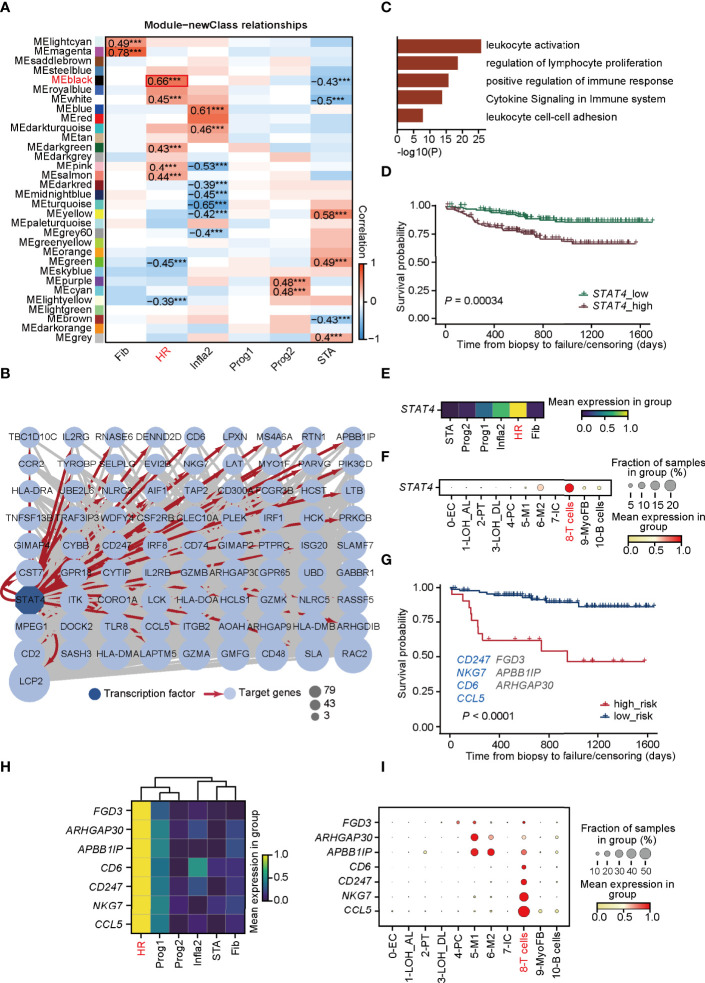
*STAT4* is essential in mediating T-cell immune responses towards HR rejection. **(A)** Heatmap presenting the 31 clusters of HVGs and the correlation between gene modules and rejection states. ***p-value < 0.001. **(B)** Visualization of co-expression network and hub genes regulated by *STAT4* from MEblack module in HR. Dark blue-filled octagonal nodes: transcription factors; light blue-filled circular nodes: target genes; red lines with arrows: regulatory relationship; node size: degree of transcription factor-target connectivity. **(C)** The enriched Gene Ontology terms for *STAT4* and its downstream regulatory genes. **(D)** The survival curves for *STAT4* in patients with renal transplantation rejection. **(E)** Matrix plot showing the expression level of *STAT4* in various rejection states. **(F)** The expression of transcription factor *STAT4* in various cell types. **(G)** The survival curve of validation cohorts with predicted high and low risk of graft failure. Genes marked in blue are known to be relevant to graft rejection and those in gray were newly identified. **(H)** Matrix plot showing the expression level of optimal genes in rejection states. **(I)** The expression of the optimal genes in various cell types. LOH_AL, loop of Henle, ascending limb; LOH_DL, loop of Henle, distal limb; Endo, endothelial cell; PT, proximal tubule; PC, principal cell; MyoFB, myofibroblast; IC, intercalated cell. HR, high-risk state; Prog1, progressive state 1; Prog2, progressive state2; Infla2, inflammatory state 2; STA, stable state; Fib, fibrosis state.

To explore the potential molecular mechanisms mediated in T cells that lead to HR rejection, WGCNA (33) was performed to seek out the gene co-expression networks (**Supplementary Figures 5A–C**). Eighteen gene modules (labeled with colors, such as MElightcyan) were generated by calculating the correlation between total genes and the allograft samples (**Supplementary Figure 5B**). The number of significant correlation coefficients between gene modules and the newly defined rejection states was far greater than the classification based on traditional clinical diagnoses, suggesting the power of this new classification (**Figure 3A**; **Supplementary Figure 5C**). Additionally, the gene module MEblack has the biggest correlation with the HR state (**Figure 3A**). To focus on modules most relevant to HR, we selected MEblack and identified that a large majority of its hub genes were regulated by signal transducer and activator of transcription 4 (*STAT4*), which was detected by IRegulon (35) (**Figure 3B**; **Supplementary Figure 6**). GO analysis of these hub genes regulated by *STAT4* showed highly activated immune functions, including leukocyte activation and regulation of lymphocyte proliferation (**Figure 3C**), indicating that *STAT4* mediated high levels of immune responses in HR rejection. Indeed, upon analyzing an external microarray dataset GSE21374 (45) from renal allograft, we observed that patients with a higher expression of *STAT4* showed poorer allograft survival in renal transplantation (**Figure 3D**). Intriguingly, *STAT4* was not only strikingly expressed in HR (**Figure 3E**), but also significantly enriched in T cells, illustrating that *STAT4* mainly conducted HR rejection in T cells (**Figure 3F**). It is well documented that *STAT4* is a member of the STAT family, which are identified as the major components of DNA-binding proteins that activate gene transcription in response to a variety of cytokines (46, 47). It contributes to the differentiation and proliferation of both Th1 and Th17 cells, which are also crucial effectors in chronic inflammatory disorders (48). Therefore, highly correlated with the development of autoimmune diseases (47), *STAT4* has a large potential to be a key regulator of graft-rejection activation.

We next investigated whether hub genes regulated by *STAT4* in T cells could contribute to allograft failure. By applying LASSO logistic regression on the randomly selected samples from GSE21374, which were regarded as the training cohorts, seven potential targets (*CD247*, *NKG7*, *CD6*, *CCL5*, *FGD3*, *APBBAIP*, and *ARHGAP30*) from the hub gene sets were determined and used to establish a diagnostic model (**Supplementary Figure 7A;** **Supplementary Table 5**). The diagnostic ability of the model was further tested in the training cohorts (**Supplementary Figures 7A–C**), and in the rest of the samples of GSE21374, which was determined as the validation cohort (**Figure 3G**), of which the ROC curves and the overall survival analysis showed a high diagnostic and prognostic power of the model. Among the seven genes, *CD247*, *NKG7*, *CD6*, and *CCL5* were presented relevant to transplantation rejection, reflecting poorer allograft survival after renal transplantation (49–52). Intriguingly, all seven genes were remarkably expressed in HR especially *CD6*, *CD247*, *NKG7*, and *CCL5*, which were specifically expressed in T cells (**Figures 3H, I**). These results revealed that *STAT4* as a core transcription factor, mediated T-cell immune responses, which is essential in HR progression and renal allograft failure.

### *PTPN6* Is a Novel Signaling Molecular Inducing *STAT4* Signaling in T Cells

**Figure 4:**
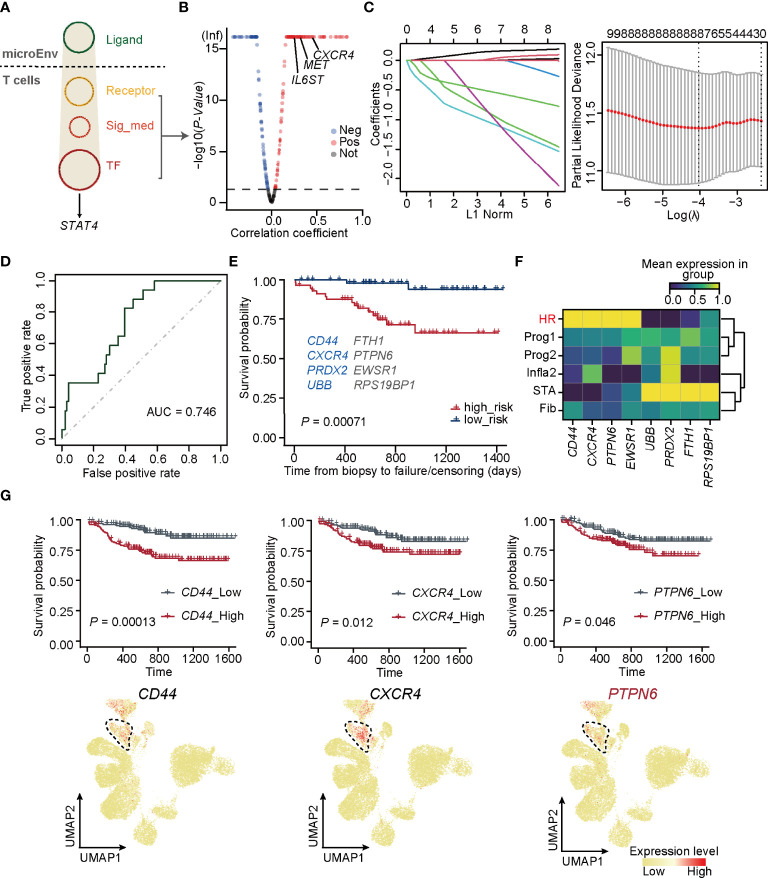
*PTPN6* is a novel regulator inducing *STAT4* signaling in T cells. **(A)** Upstream regulatory networks targeting of *STAT4* in T cells form HR. Sig_med, signaling mediator; TF, transcriptional factors; MicroEnv, microenvironment. **(B)** Scatter plot showing negative and positive correlations between *STAT4* and its upstream-regulators. The red, blue, and gray dots indicate upstream regulators that were considered to be positive, negative, and no correlation, respectively. **(C)** The coefficient plot of the LASSO model (left panel) and the selection of the tuning parameter in LASSO logistic regression analysis (right panel). **(D)** ROC curves for allograft loss diagnosis prediction in the validation cohorts. € The survival plot of validation cohorts with predicted high and low risk of graft failure. Genes marked in blue are known to be relevant to graft rejection and those in gray were newly identified. **(F)** Matrix plot showing the expression level of optimal genes in each rejection state. **(G)** The survival plot (top panel) and UMAP plot (bottom panel) of *CD44*, *CXCR4*, and *PTPN6* showing the relative expression level in each rejection state.

Since *STAT4* and almost all of its putatively target genes triggered adverse allograft survival in patients who received renal transplantation, the essential upstream regulators of *STAT4* deserve further identification. To explore upstream signaling pathways targeting *STAT4*, Nichenet’s (36) analysis was first performed to determine the overall ligand and receptor pairs targeting *STAT4* in T cells from the HR group (**Figure 4A**). All of the receptors, signaling mediators, and transcription factors (TFs) in the network were picked and those uncorrelated to HR were filtered out by limiting the *p*-value larger than 0.05, of which *IL6ST*, *MET*, and *CXCR4* were verified as upstream signaling molecules to regulate *STAT4* (53–55) (**Figure 4B**). We applied these signaling molecules to the LASSO logistic regression on the training cohorts, which were randomly selected from GSE21374, and eight optimal genes, namely, *CD44*, *FTH1*, *CXCR4*, *PTPN6*, *PRDX2*, *EWSR1*, *UBB*, and *RPS19BP1*, were employed to establish a diagnostic model (**Figure 4C**; **Supplementary Table 5**). ROC curves and overall survival analysis revealed a high diagnostic and prognostic power of the model on both the training cohorts (**Supplementary Figure 8A**) and the validation cohorts, which consisted of samples in GSE21374 excluding those in the training set (**Figure 4D**). Among these genes, *CD44*, *CXCR4*, *PRDX2*, and *UBB* were significantly related to transplantation rejection and poor survival (**Figure 4E**), which were also proved by researchers’ studies (56–59). The other four were newly discovered genes potentially playing key roles in rejection and graft failure (**Figure 4E**). Interestingly, we found that *CD44*, *CXCR4*, *PTPN6*, and *EWSR1* were specifically expressed in an HR state from the new classifications in bulk RNA-seq (**Figure 4F**). As expected, the overall survival probability of patients with higher expression of *CD44*, *CXCR4*, and *PTPN6* showed worse disease consequences (**Figure 4G**), suggesting that these genes were HR regulators of allograft loss. It is known that antagonists of *CD44* and *CXCR4* can help improve outcomes in allograft rejection (56, 60), which further supports our hypothesis.

Importantly, *PTPN6*, which was newly identified by our gene regulatory network, encodes a member of the protein tyrosine phosphatase (PTP) family and regulates multiple cellular processes, such as cell growth, differentiation, and tumorigenesis (61). It is demonstrated that *PTPN6* functions in a TCR-dependent manner (62) and elevated expression of *PTPN6* recruits infiltration of T cells (63), illustrating its role in mediating *STAT4*-induced T-cell immune responses.

### External Data Re-Confirm the Power of Our New Classification Strategy and *STAT4*-Mediated Allograft Loss in HR Rejection

**Figure 5:**
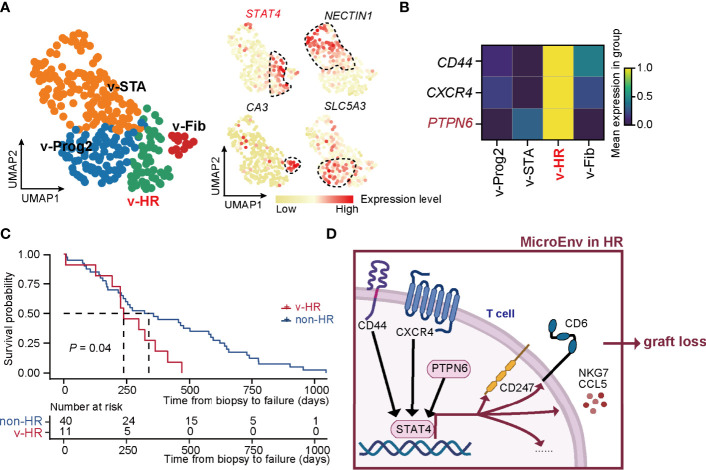
New classification pipeline re-confirms *STAT4*-mediated allograft loss in HR rejection. **(A)** UMAP plots showing the validation dataset and its annotation based on the marker genes of rejection states. The black dashed box represents the corresponding state. **(B)** Matrix plot showing the expression level of *CD44*, *CXCR4*, and *PTPN6* in each rejection state. **(C)** The survival curve of patients with HR versus non-HR rejection of renal transplantation. **(D)** Diagram of renal allograft failure triggered by HR rejection that is mediated by *PTPN6*-*STAT4*-immune responsive signaling in T cells. MicroEnv, microenvironment.

To confirm the precision of the unsupervised classification built in our study, we applied this strategy to the external microarray dataset (GSE21374) and yielded 4 rejection states (**Figure 5A**). Each status was finely characterized and annotated by the signature gene sets, including STA (v-STA, *NECTIN1* ^+^), Fib (v-Fib, *CA3* ^+^), Prog2 (v-Prog2, *SLC5A3* ^+^), and HR of validation (v-HR, *STAT4* ^+^). Therefore, both the method and signature genes identified in rejection states worked well in these external data. Importantly, *PTPN6*, as well as *CD44* and *CXCR4*, were upregulated specifically in HR in the validation cohort, demonstrating that the two known regulators, especially the newly discovered gene *PTPN6*, were critical in HR progression (**Figure 5B**).

To further confirm the HR characteristic prone to allograft loss in v-HR, we isolated samples with a final diagnosis of allograft loss, and divided them into non-HR (not belonging to v-HR) and v-HR according to the new classification. Indeed, survival probability analysis revealed that patients from v-HR showed greater susceptibility to renal failure compared to the non-HR group (**Figure 5C**).

Taken together, our new classification strategy proves to be precisely helpful for distinguishing renal allograft rejection status, upon which we also provide new insights into *PTPN6*-*STAT4*-immune responsive signaling in T cells that mediates HR rejection (**Figure 5D**).

## Discussion

Despite the histology-dependent diagnosis of renal allograft rejection being the currently widely accepted criterion, the limitations in precisely defining pathogenesis remain to be an obvious clinical concern. To uncover the molecular mechanisms of renal allograft rejection in an easier, more precise and high-throughput manner, three key pieces of information are required: (1) transcriptome-based accurate classification with high universality and easy operation, (2) identification of key cell types driving rejection progression, and (3) well-documented combination between bulk transcriptomic and scRNA-seq data. Here, by reclassifying renal allograft rejection state based on an unsupervised pipeline, we uncovered an HR rejection status prone to allograft loss and revealed that T-cell immune responses mediated by *PTPN6*-*STAT4* signaling were essential in triggering allograft failure.

Based on the unsupervised algorithms, the new classification pipeline avoids the mismatched pathogenesis with clinical diagnoses and reveals different rejection states at the transcriptomic level, especially the focused HR state. To our knowledge, the HR state is a newly discovered stage predominantly correlated with graft-versus-host disease and induces immune activation responses, which we consider to be prone to allograft failure. This is further proved by recent lines of evidence from renal transplantation showing that allograft failure is highly associated with prolonged immune activation (64, 65). HR mainly recruited T-cell and B-cell effector transcripts to active mixed rejection, but is even more probable to cause graft loss than pure ABMR or TCMR (66). It is reported that the number and function of T cells are always considered being inhibited at an early stage of renal transplantation by immunosuppressive drugs (67). T-cell depletion eliminates anti-donor alloantibodies and conferred protection from destruction of renal allografts (68, 69). Therefore, HR is reasonable to bear more burden from cytotoxic lymphocytes and effector T cells. Taken together, activated T-cell immune responses that re-aggregate significantly in HR rejection will most likely drive graft failure.

Mechanistically, we found that *STAT4* is essential to stimulate T-cell activation (**Figure 5D**). Apart from the known regulators *CD44 (* 56) and *CXCR4* (57), we also newly identified *PTPN6*, which is associated with tumor rejection (61, 63) and T-cell aggregation (62, 63), to be essential in promoting renal transplantation rejection. *PTPN6* functions as an upstream regulator to activate *STAT4* and further impel the downstream immune gene set signaling, including activation of receptors and signaling mediators (49, 51), as well as the release of different kinds of cytokines (50, 52). Ultimately, allograft failure is inevitable due to the continuous and uncontrollable accumulation of inflammatory burden derived from T cells.

## Conclusion

Our work provides a new classification for renal transplant rejection at the systemic transcriptomic level, along with corresponding signature genes and cell types. We also propose an important rejection state HR, which is most prone to allograft loss and highlights *PTPN6*-*STAT4*-proinflammation signaling in T cells, which plays critical roles in triggering allograft failure. This proposed strategy together with a new pathogenic mechanism provides a new path for potential clinical diagnosis and intervention for renal transplantation rejection.

## Data Availability Statement

The datasets presented in this study can be found in online repositories. The names of the repository/repositories and accession number(s) can be found in the article/**Supplementary Material**.

## Author Contributions

YC, BZ, TL, and HZ: study design. YC: sample and data acquisition. YC and XC: data analysis. YC: drafting of the manuscript. BZ, YW, and HZ: revising of the manuscript. All authors contributed to the article and approved the submitted version.

## Funding

This work was supported by the Science and Technology Program of Guangzhou (grant number: 202002030429), the National Natural Science Foundation of China (grant numbers: 32000840 and 31871370), and the National Key R&D Program (grant number: 2019YFA0801703).

## Conflict of Interest

The authors declare that the research was conducted in the absence of any commercial or financial relationships that could be construed as a potential conflict of interest.

## Publisher’s Note

All claims expressed in this article are solely those of the authors and do not necessarily represent those of their affiliated organizations, or those of the publisher, the editors and the reviewers. Any product that may be evaluated in this article, or claim that may be made by its manufacturer, is not guaranteed or endorsed by the publisher.

## Abbreviations

HR, high-risk; ESKD, end-stage kidney disease; scRNA-seq, single-cell RNA sequencing; GEO, Gene Expression Omnibus; STA, stable state; ABMR, antibody-mediated rejection; TCMR, T-cell-mediated rejection; Mix, Mixed state, ABMR and TCMR; AR, acute rejection; CR, chronic rejection; GSE, series record of GEO; HVGs, highly variable genes; UMAP, Uniform Manifold Approximation and Projection; WGCNA, weighted correlation network analysis; DEGs, differentially expressed genes; GO, Gene Ontology; Fib, fibrosis state; Infla1/2, inflammatory state 1/2; Prog1/2, progressive state 1/2; M1/2, type I/II macrophages; LASSO, least absolute shrinkage and selection operator; PTP, protein tyrosine phosphatase; v-STA/Fib/Prog2/HR, STA/Fib/Prog2/HR of validation; STAT4, signal transducer and activator of transcription 4.
