# Supplementary material for: T Cells With Activated STAT4 Drive the High-Risk Rejection State to Renal Allograft Failure After Kidney Transplantation
Source: Front Immunol. 2022 Jul 1;13:895762. doi: 10.3389/fimmu.2022.895762 (PMC9283858; doi:10.3389/fimmu.2022.895762)
Supplement: Supplementary file 1 [file DataSheet_1.pdf]

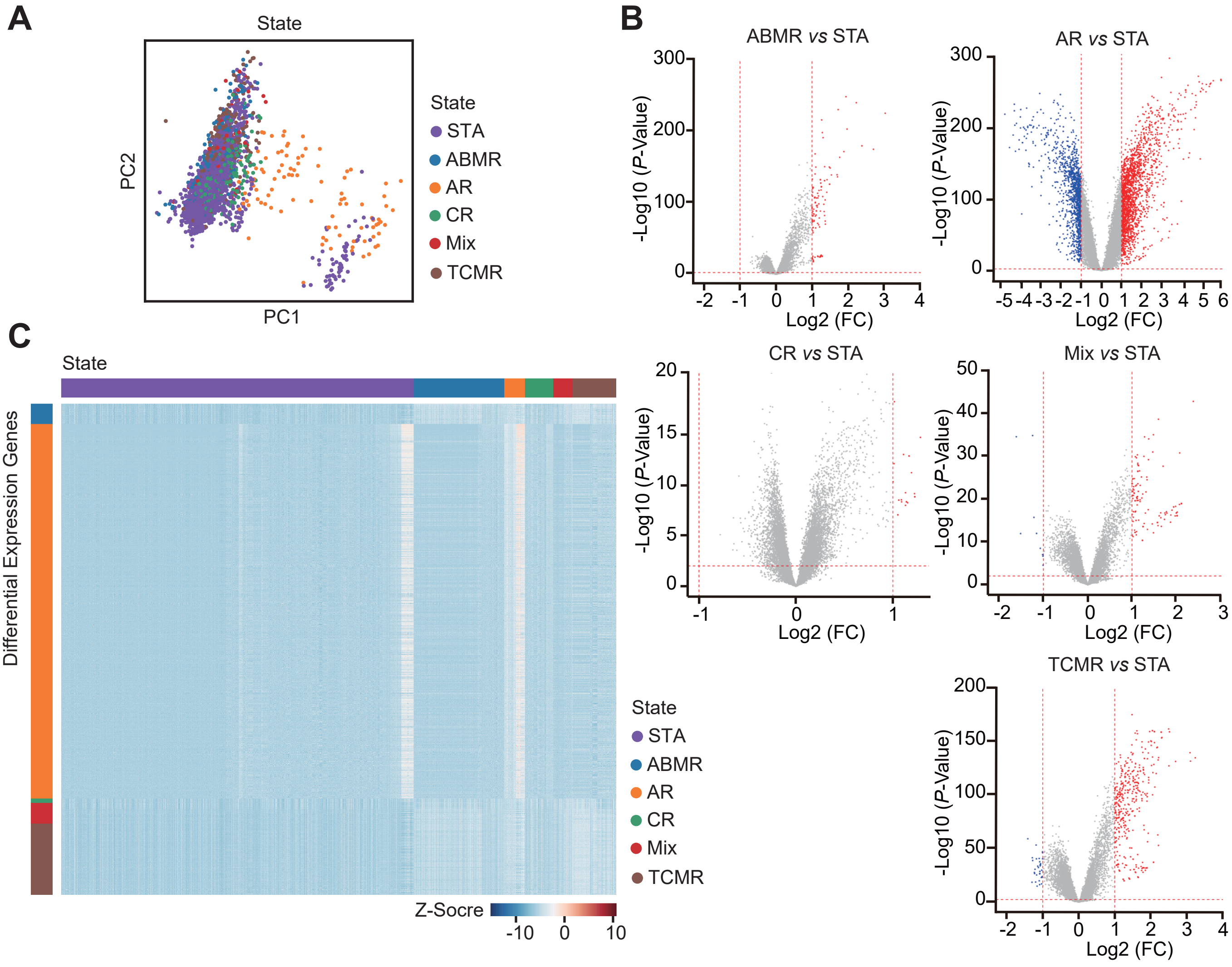

Supplementary Figure 1. Traditional clinical histological classification is limited in diagnosis of renal allograft failure.

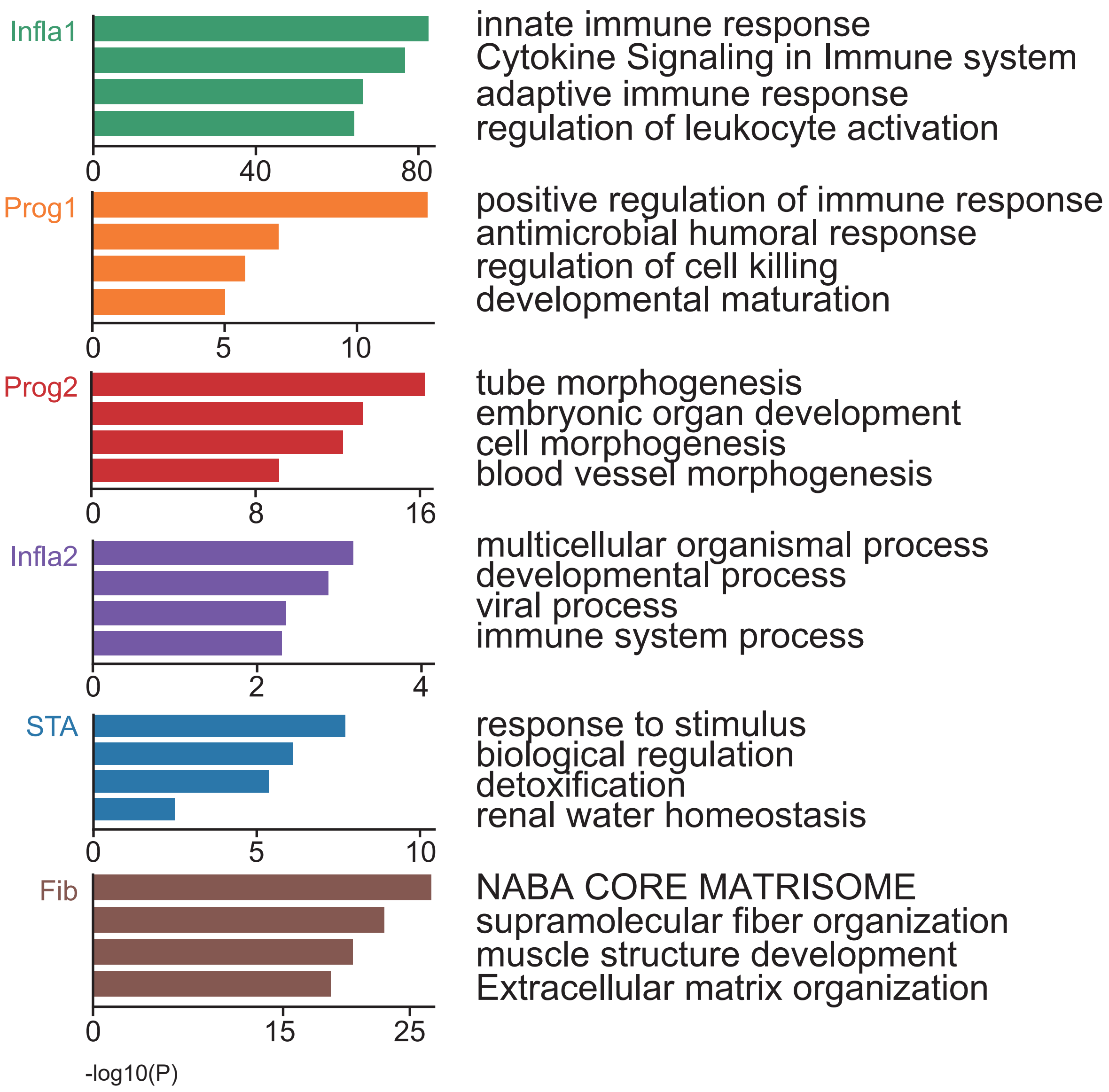

Supplementary Figure 2. The enriched GO terms for the corresponding rejection state.

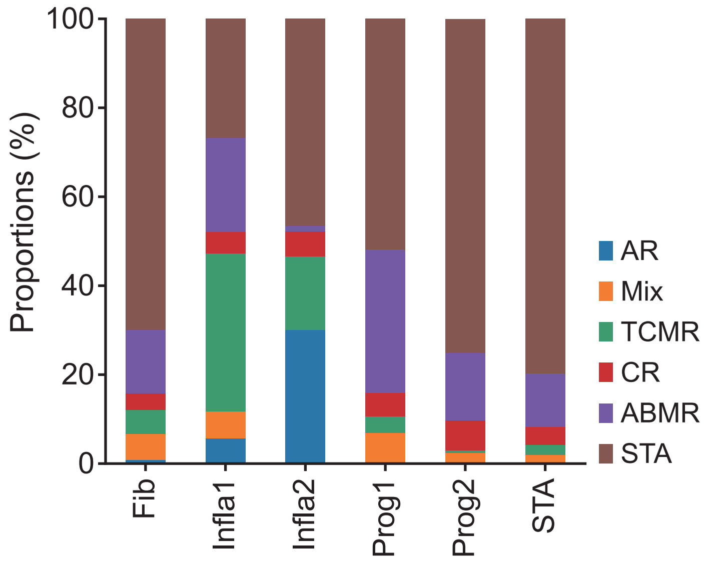

Supplementary Figure 3. Proportions of renal allograft samples diagnosed with traditional criterion in each of the new rejection classifications.

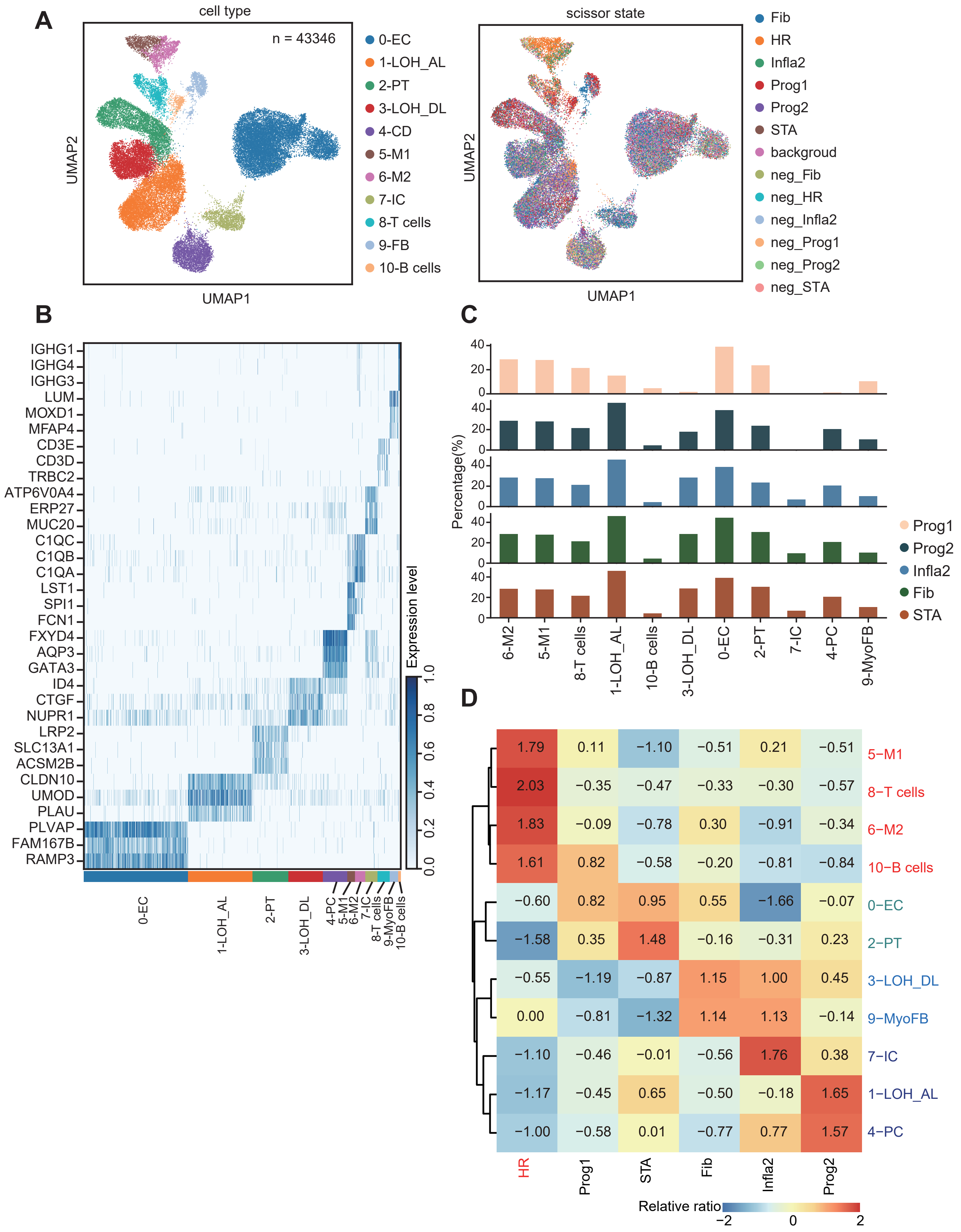

Supplementary Figure 4. Immune cells were enriched in HR rejection at single cell resolution.

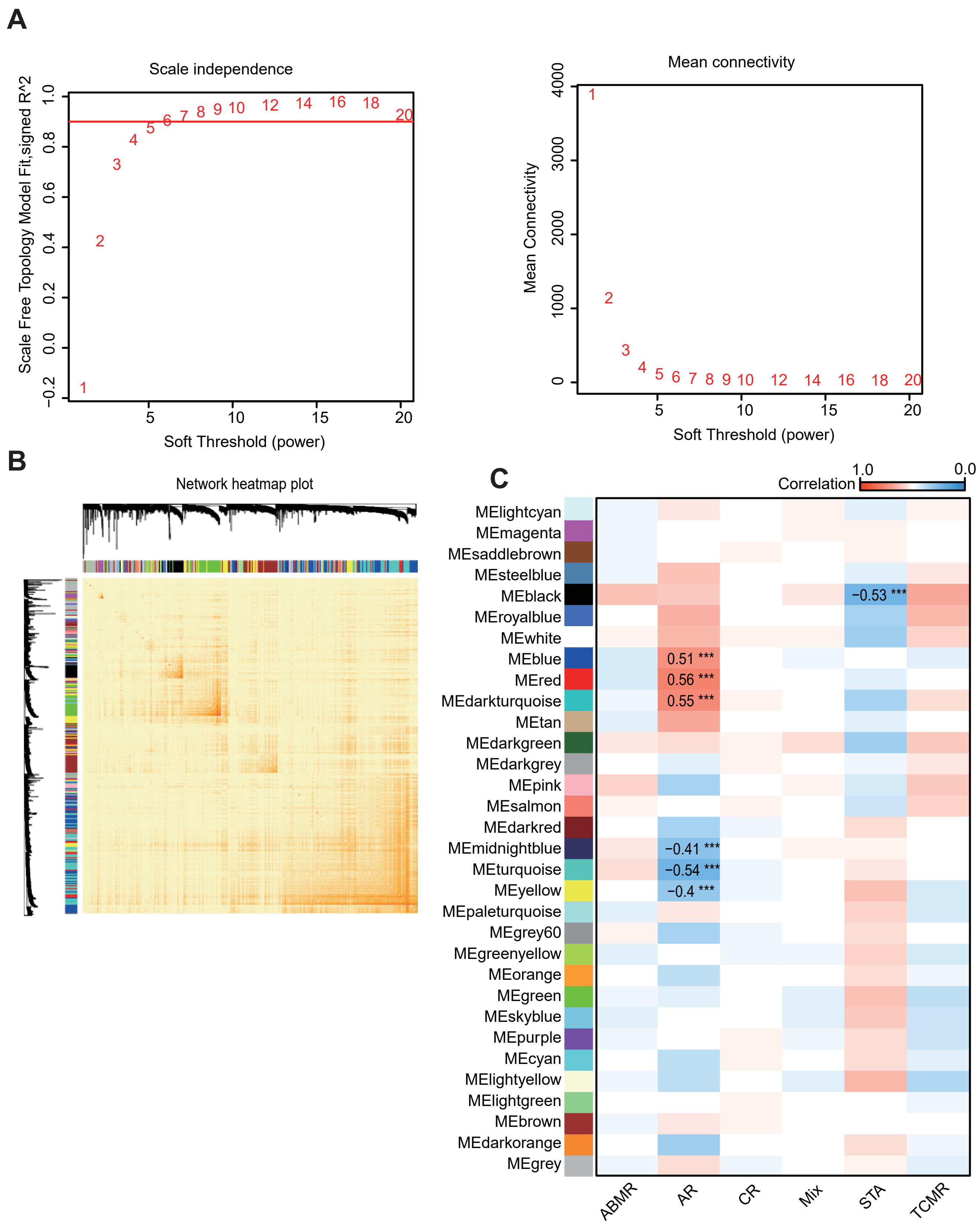

Supplementary Figure 5. Gene co-expression networks based on gene expression patterns and traditional diagnosis classification.

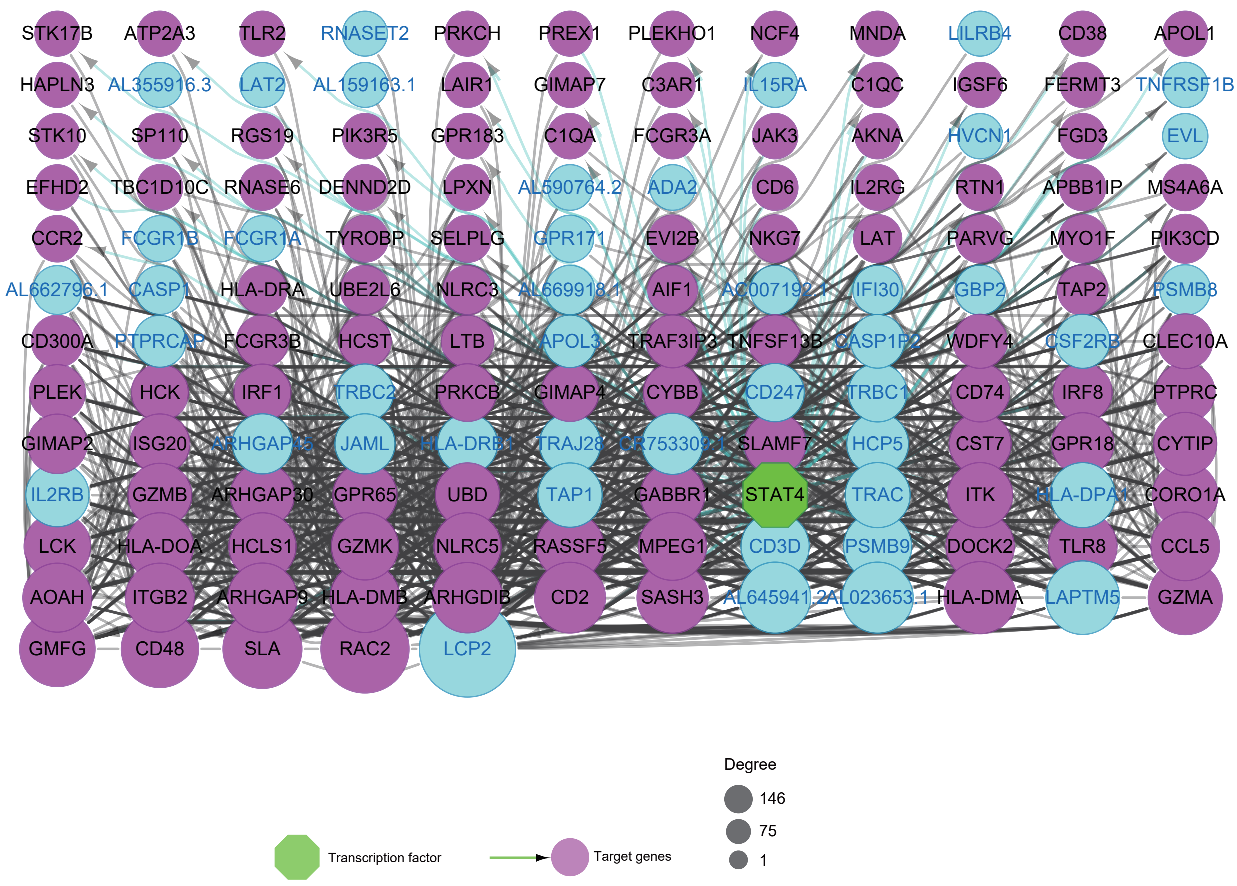

Supplementary Figure 6. Visualization of co-expression network and hub genes from MEblack module in HR.

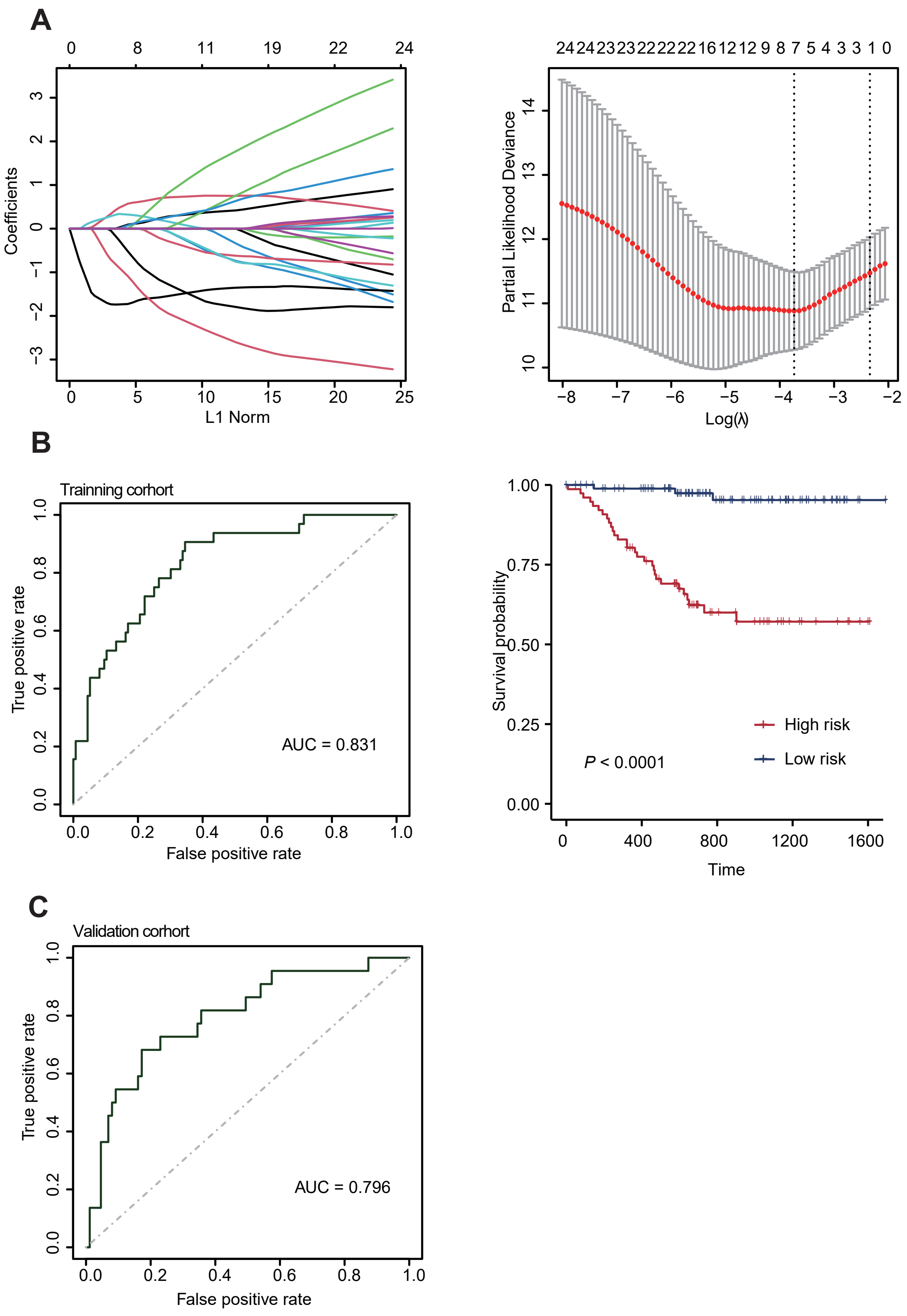

Supplementary Figure 7. Establishment and validation of diagnostic model.

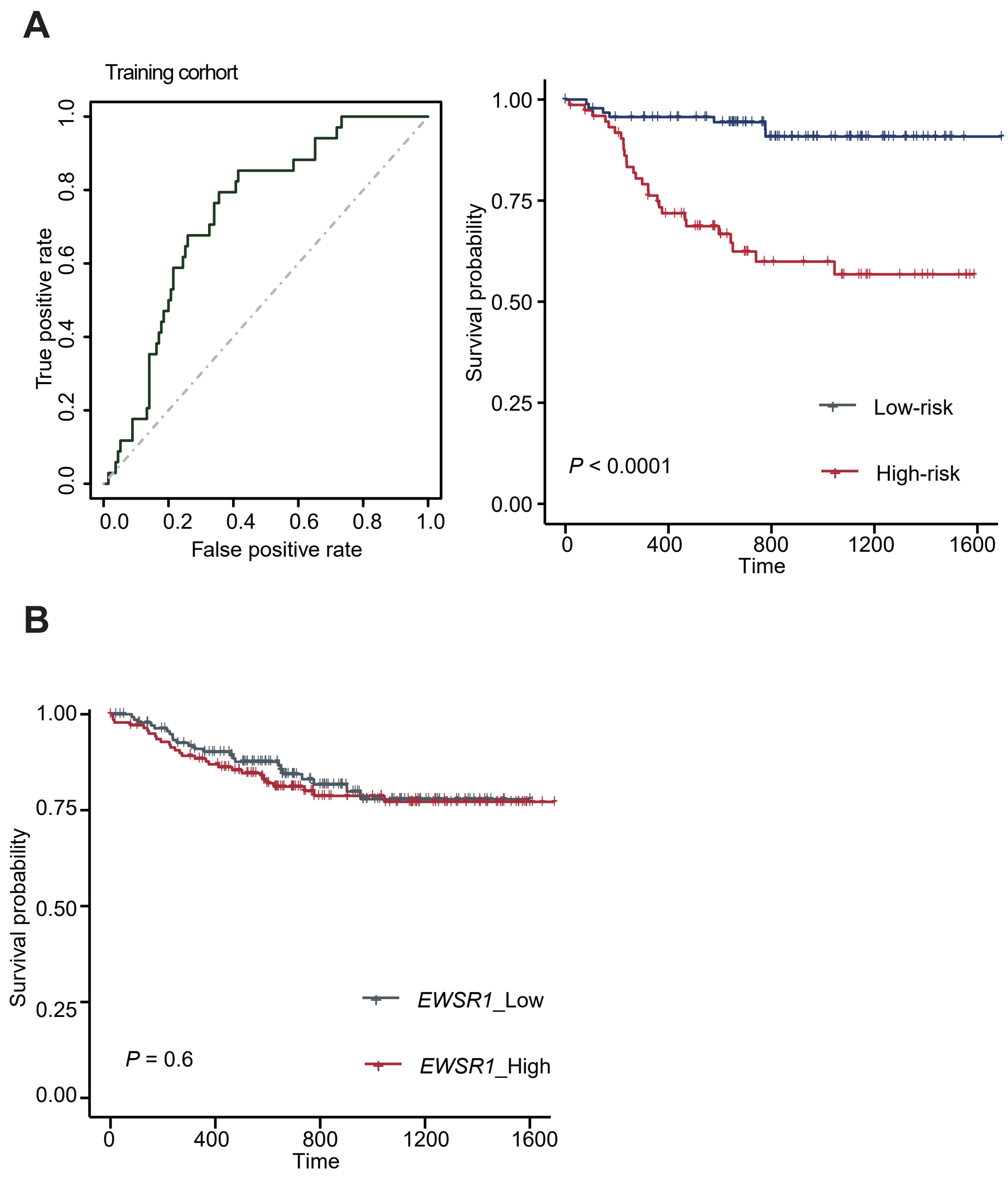

Supplementary Figure 8. Establishment and validation of diagnostic model.

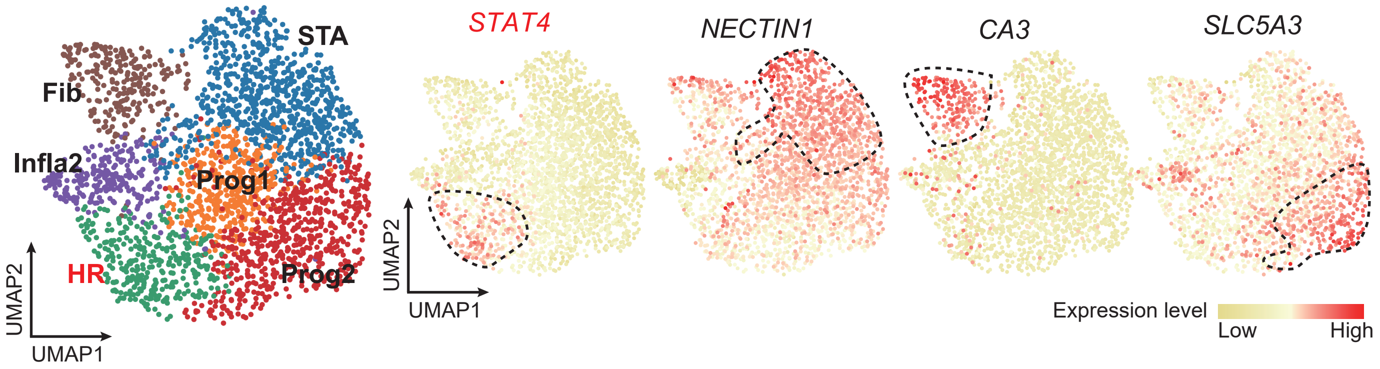

Supplementary Figure 9. UMAP plots showing the validation set was annotated based on the marker genes of rejection states, and the black dashed box represents the corresponding states.
